# MicroAge mission: experimental design and hardware for a bespoke culture system supporting tissue-engineered skeletal muscle

**DOI:** 10.1038/s41526-026-00579-z

**Published:** 2026-02-21

**Authors:** Samantha W. Jones, Shahjahan Shigdar, Jonathan Temple, Benjamin Tollitt, Adam Janvier, Fiona Mutter, James R. Henstock, Jessica Ohana, David A. Turner, Christopher McArdle, Gianluca Neri, William Blackler, Georgi Olentsenko, Kai. F. Hoettges, Anne McArdle, Malcolm J. Jackson

**Affiliations:** 1 Department of Musculoskeletal and Ageing Science, Institute of Life Course and Medical Sciences, Faculty of Health and Life Sciences, University of Liverpool, Liverpool, UK; 2 MyoLine Platform, Sorbonne Université, Inserm, Institut de Myologie, Centre de Recherche en Myologie, Paris, France; 3 Kayser Space Ltd, Rutherford Appleton Laboratory Building, Harwell Campus, UK; 4 School of Electrical Engineering, Electronics and Computer Science, Department of Electrical Engineering and Electronics, University of Liverpool, Liverpool, UK; 5 Present Address: Department of Applied Sciences, Faculty of Health & Life Sciences, University of Northumbria, Newcastle-upon-Tyne, UK

**Keywords:** Biological techniques, Biophysics, Biotechnology, Cell biology, Engineering, Physiology

## Abstract

Microgravity provides a unique model for accelerated skeletal muscle loss and potentially muscle ageing. During spaceflight, astronauts experience pronounced muscle atrophy, similar to age-related decline on Earth but over a much shorter timescale. Despite daily aerobic and resistance exercise on the International Space Station (ISS), countermeasures remain suboptimal, reflecting similar challenges seen in ageing populations. The MicroAge Mission used microgravity on the ISS to assess whether the molecular mechanisms behind reduced adaptive responses to contractile activity during ageing resemble those triggered by spaceflight. It also tested proof-of-concept genetic interventions, including Heat Shock Protein 10 (HSP10) overexpression, to mitigate muscle atrophy and functional loss. A tissue-engineering approach was used to fabricate human skeletal muscle constructs secured to 3D-printed scaffolds. These scaffolds incorporated microfluidic channels to interface with the flight hardware’s fluid-handling system. The hardware, developed by Kayser Space Ltd, was designed to operate with the European Space Agency’s (ESA) Kubik incubator on the ISS. This research addresses key methodological constraints in low Earth orbit (LEO) experimentation, outlining pre-flight protocol development, muscle construct biofabrication methods, and operational considerations. The findings provide a translational framework for future studies on musculoskeletal degeneration, with implications for therapies targeting both terrestrial ageing and astronaut musculoskeletal health.

## Introduction

Microgravity potentially offers a powerful model for understanding accelerated skeletal muscle ageing, providing insights into the molecular mechanisms underlying muscular decline^1^. During spaceflight, astronauts experience rapid reductions in skeletal muscle mass, with virtually all muscle groups showing a 3–10% decrease in volume after just 17 days in microgravity^2–4^. Prolonged exposure (≥6 months) exacerbates these effects, leading to significant physiological changes that appear to mirror age-related muscle decline on Earth^4^. Similar to ageing, microgravity affects specific muscle groups disproportionately, though all fibre types are ultimately compromised^3,5^. Despite the implementation of daily (~2 h/day) aerobic and resistance exercise on the International Space Station (ISS), the preventative effects remain incomplete, echoing the challenges of exercise efficacy in ageing populations^6,7^.

On Earth, demographic shifts driven by declining fertility and mortality rates have resulted in a growing number of older adults (aged ≥65) with relatively poor health and diminished quality of life^8^. Age-related loss of skeletal muscle mass and function, a major contributor to physical frailty, significantly increases the risk of falls and hospitalisation. By the age of 70, muscle cross-sectional area can decrease by ~25–30%, with muscle strength diminishing by ~30–40%, accompanied by muscle atrophy and weakened fibres^9–11^.

In healthy adults, contraction-mediated biochemical responses to exercise drive anabolic changes in skeletal muscle. These adaptive changes are initiated by an acute increase in reactive oxygen species (ROS), which act as redox-signalling molecules under normal physiological conditions^12–14^. However, ageing is associated with attenuated ROS-mediated adaptive responses, reducing exercise efficacy. This decline is thought to stem from chronically elevated mitochondrial H_2_O_2_ production^9,15,16^.

The MicroAge Mission aimed to leverage microgravity conditions on board the ISS to investigate whether the molecular mechanisms driving diminished adaptive responses to contractile activity in ageing are the same as those observed during spaceflight. This research holds promise for advancing our understanding of skeletal muscle decline and developing effective interventions for both ageing populations and astronauts^1^.

In addition to the primary objectives, the mission sought to investigate proof-of-concept genetic intervention strategies for maintaining muscle function, specifically through the overexpression of Heat Shock Protein 10 (HSP10), a mitochondrial chaperone known to offer some protection against loss of maximal tetanic force and loss of muscle cross-sectional area observed in old mice^17^. The study also evaluated the impact of simulated Earth gravity (1 g) using centrifugation, alongside a comprehensive post-flight ground reference experiment (GRE) to generate appropriate ground-based control data.

This paper presents the experimental design and hardware development undertaken for the MicroAge mission, with the aim of supporting researchers conducting biological investigations in space. It details the rationale behind the flight hardware design, pre-flight planning, muscle construct biofabrication techniques, and operational considerations specific to low Earth orbit (LEO) research. By sharing practical insights and lessons learned, the paper offers a framework that can be adapted for future missions, helping to streamline and standardise workflows for space-based life science research.

To enable the MicroAge studies, a tissue-engineering strategy was employed to generate human skeletal muscle constructs anchored to custom-designed 3D-printed scaffolds. These scaffolds featured integrated microfluidic channels that interfaced with a specialised fluid handling system housed within the flight hardware. The hardware units, developed by Kayser Space Ltd, were specifically engineered for compatibility with the European Space Agency’s (ESA) Kubik incubator, located in the Columbus module of the ISS.

Conducting biological experiments in LEO presents a range of methodological and logistical challenges. Although the ISS has supported cell-based life science research for over two decades, variability remains in experimental protocols, resource availability, and equipment standardisation^18^. By documenting the MicroAge mission’s approach, this paper contributes to a growing body of knowledge aimed at improving consistency and efficiency in future spaceflight experiments.

## Methods

### Materials

Human immortalised myoblasts (Lot AB1167: *fascia lata* biopsy, 20 years, male), generated using the MyoLine Platform, were obtained via a Materials Transfer Agreement (MTA) from the Institute of Myology (Paris, France)^19,20^. Skeletal muscle cell growth medium kits (phenol red-free) were obtained from PromoCell (Dorset, UK).

High glucose, Dulbecco’s Modified Eagle Medium (DMEM) with GlutaMAX™ (±HEPES buffer), Leibovitz L-15 medium with GlutaMAX™, Gibco CO_2_ independent medium, RNA*later*™, foetal bovine serum (FBS), horse serum, goat serum, LIVE/DEAD™ viability kit, Alexa Fluor-488 nm Phalloidin, Alexa Fluor-488 nm goat anti-rabbit secondary antibody, DAPI solution (1 mg/mL)and ProLong™ gold antifade mountant were purchased from ThermoFisher Scientific (Altrincham, UK).

The flight hardware and associated consumables were designed, manufactured or procured by Kayser Space Ltd (Didcot, UK). Unless stated otherwise, all other chemicals used in this study were obtained from Sigma Aldrich, Dorset, UK.

### Cell culture

Human immortalised myoblasts (AB1167_CTL) were routinely maintained in a complete growth medium consisting of PromoCell skeletal muscle basal medium (phenol red-free) supplemented with 20% (v/v) FBS, 50 μg/mL bovine fetuin, 10 ng/mL epidermal growth factor, 1 ng/mL basic fibroblast growth factor, 10 μg/mL recombinant human insulin, 0.4 µg/mL dexamethasone, 10 μg/ mL gentamicin and 2 mM L-glutamine^19,20^.

Cells were incubated in a humidified environment at 37 °C with 5% (v/v) CO_2_; medium exchanges were performed every 48 h. All stock cultures (AB1167_CTL) were maintained at <70% confluence and used between passages 4–8 to prevent visual decline of spontaneous contractions.

### Biofabrication of human skeletal muscle constructs

**Fig. 1:**
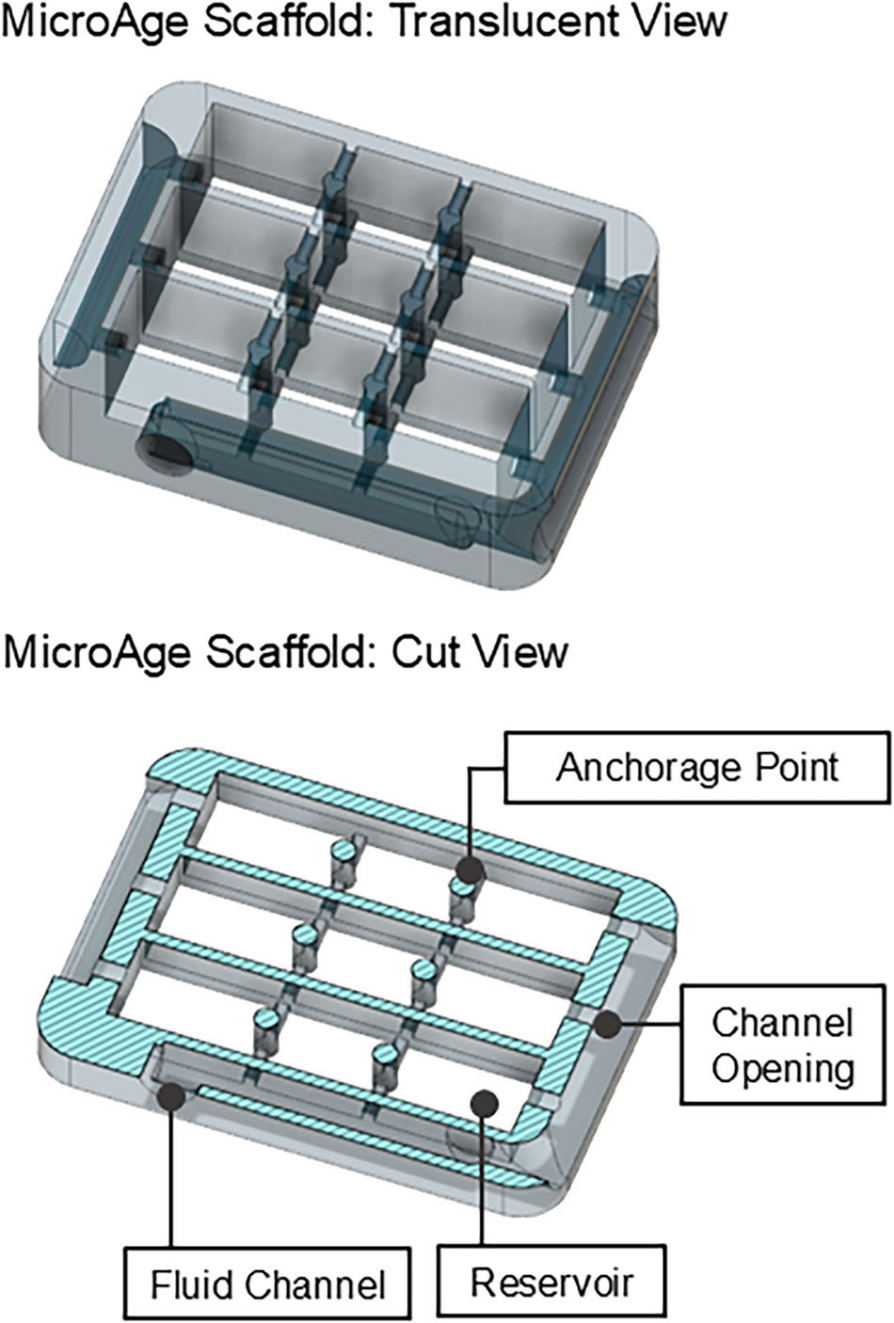
Computer Aided Design (CAD) rendering of the MicroAge scaffold design. The figure illustrates the internal channel architecture, cell seeding reservoirs and anchorage points. Images were produced using AutoCAD.

**Fig. 2:**
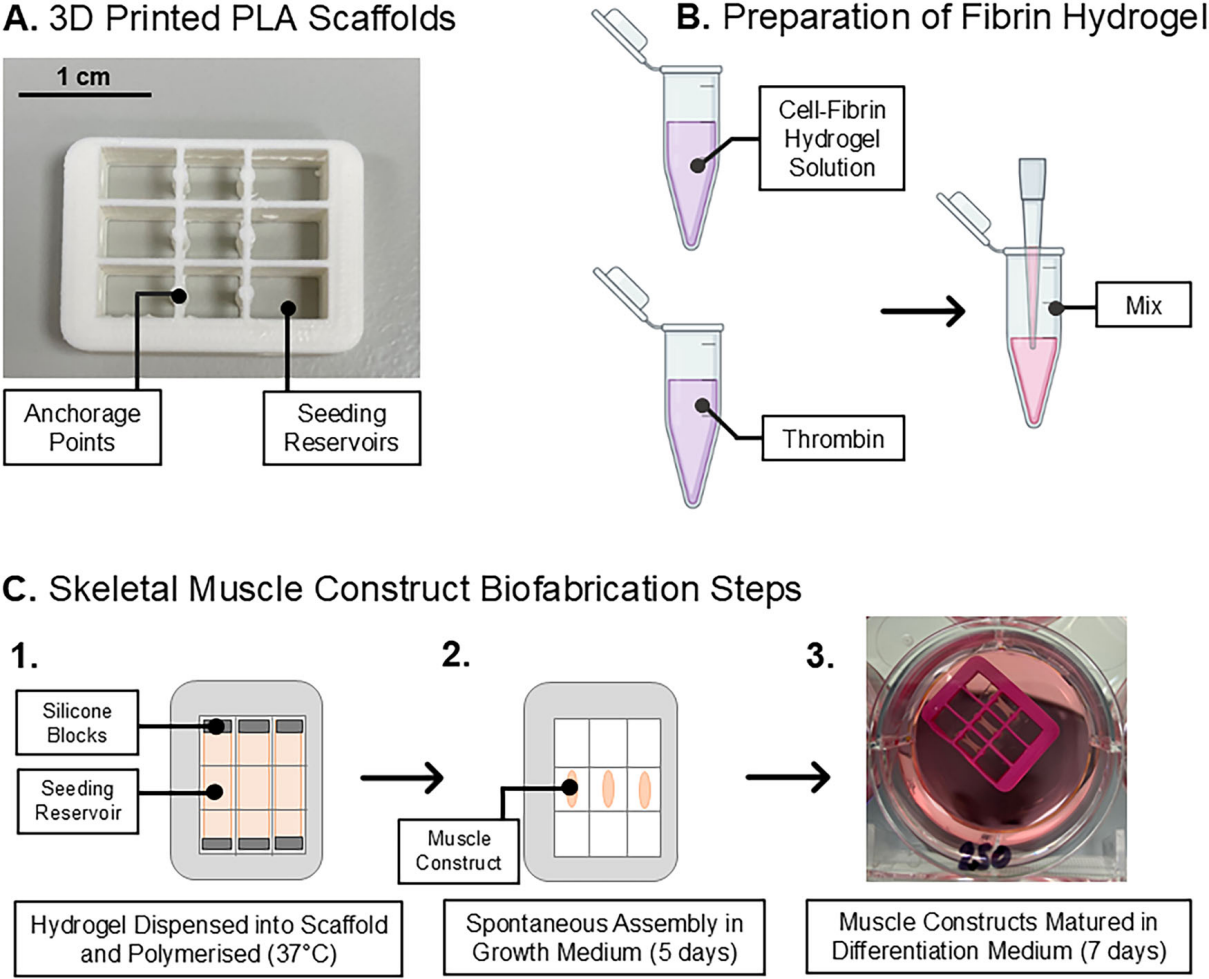
Schematic diagram of the muscle construct biofabrication process. **A** Biocompatible scaffolds were generated by fused filament fabrication on an UltiMaker 2+ instrument. **B** Myoblasts were dissociated by trypsinisation to produce a single-cell suspension before being mixed with a fibrin-hydrogel solution. **C** The hydrogel solution was dispensed into the scaffolds and polymerised at 37 °C for 20 min. The muscle constructs were allowed to spontaneously assemble around the scaffold anchor points over 5 days, after which they underwent maturation for a further 7 days.

Bespoke, biocompatible scaffolds were produced by fused filament fabrication (FFF) 3D printing on an UltiMaker 2^+^ instrument with polylactic acid (PLA) filament. The design solution included internal channels that integrated with the fluid handling circuit of the flight hardware, in addition to three individual reservoirs, each with two anchorage points, around which the muscle constructs spontaneously assemble (Figs. 1 and 2A). Post-print, Sylgard 184 (polydimethylsiloxane) blocks were placed at either end of the individual scaffold reservoirs to cover the openings to the internal fluidic channels. The scaffold assemblies were then sterilised in 70% ETOH before coating in sterile 0.2% Pluronics**™** F127 overnight (+4 °C).

Expanded myogenic cell populations were maintained in complete growth medium, dissociated in 1X trypsin-EDTA to a single cell suspension and encapsulated in a fibrin hydrogel solution before dispensing into the individual scaffold reservoirs at a density of 2.5 × 10^5^ cells/construct. Each individual scaffold accommodated three muscle constructs.

To prepare one scaffold, a cell solution (7.5 × 10^5^ cells in 272 μL growth media supplemented with 1.5 mg/mL aminocaproic acid (ACA)) was combined with a hydrogel solution (99 μL growth factor reduced ECM gel (20% v/v) and 87 μL fibrinogen (20 mg/mL) in a 1.5 mL polypropylene tube on ice.

To this, 37 μL bovine thrombin was added before pipetting vigorously to mix and dispensing 150 μL of the cell-hydrogel mixture into each of the three scaffold reservoirs (Fig. 2C). The cell-hydrogel mixture was polymerised at 37 °C (5% v/v CO_2_) for 20 min followed by incubation in complete growth media supplemented with 1.5 mg/mL aminocaproic acid for 5 days. During this time, the muscle constructs spontaneously assembled around the static anchorage points within the scaffolds (Fig. 2C).

Subsequently, the media was replaced with differentiation medium (DMEM with GlutaMax™ supplemented with 2% (v/v) horse serum, 10 µg/mL recombinant human insulin, 2 mg/mL ACA and 10 µg/mL gentamicin) for a further 7 days to promote differentiation of myoblasts to myotubes. Routine exchanges of differentiation medium were performed every 48 h.

Further characterisation of the model has been conducted by Tollitt et al., including quantitative proteomic profiling^21^.

### Fluorescent staining and imaging of whole muscle constructs

Whole muscle constructs were rinsed with 1× PBS and fixed in 4% paraformaldehyde (PFA) overnight at 4 °C. Brightfield images were captured on a Zeiss Axio Observer Apotome using the 2.5× objective lens. Resultant images were tiled using ImageJ.

The muscle constructs were then permeabilized in a buffer containing 0.2% (v/v) Tween-20 and 0.5% (v/v) Triton X-100 in 1X PBS for 1 h at room temperature. Following permeabilisation, constructs were incubated with AlexaFluor 488-conjugated phalloidin (1:20) and DAPI (1:1000), both diluted in the same buffer. Fluorescent imaging was performed using a Zeiss Axio Observer Apotome microscope with a 10× objective lens, using excitation/emission wavelengths of 488/496 nm (AlexaFluor 488) and 350/465 nm (DAPI).

### Cryosectioning and fluorescent staining of muscle constructs

Muscle constructs were rinsed with 1× PBS and fixed in paraformaldehyde (PFA) overnight at 4 °C, followed by sequential incubation in 10 and 30% (w/v) sucrose solutions for 24 h each. Constructs were then embedded in optimal cutting temperature (OCT) compound and snap-frozen in liquid nitrogen-cooled isopentane.

Longitudinal cryosections (10 µm thick) were prepared using a Leica Biosystems CM1950 cryostat. Sections were rehydrated in 1× PBS and subsequently blocked with a permeabilisation solution containing 0.5% (v/v) Triton X-100 and 10% (v/v) goat serum for 1 h at room temperature. Primary antibodies were diluted in 5% (v/v) goat serum in 1× PBS and applied to the sections for 1 h at 4 °C (sarcomeric α-actinin, 1:500). After washing with 1× PBS, sections were incubated with AlexaFluor 488-conjugated goat anti-rabbit secondary antibody (1:500) and DAPI (1:1000), diluted using 5% (v/v) goat serum in 1× PBS.

Sections were mounted using ProLong Gold™ antifade solution before images were captured on a Zeiss LSM 800 confocal microscope with a 40× objective lens, using excitation/emission wavelengths of 488/496 nm (AlexaFluor 488) and 350/465 nm (DAPI).

### Flight hardware design overview

The flight hardware (FH) configuration was composed of two major components; (1) an Experiment Unit (EU) including a Culture Chamber (CC) to house the muscle constructs and (2) the experiment container (EC), based on a standard Kubik Interfacing Container (KIC) footprint, providing an electrical and mechanical interface with the Kubik incubator on board the ISS. The two components together provided two layers of liquid containment (LoC) for the experiment and are described in more depth in the following sections. The materials used to manufacture the EU were selected based on their widely known biological compatibility and excellent chemical resistance.

### Experiment unit design

**Fig. 3:**
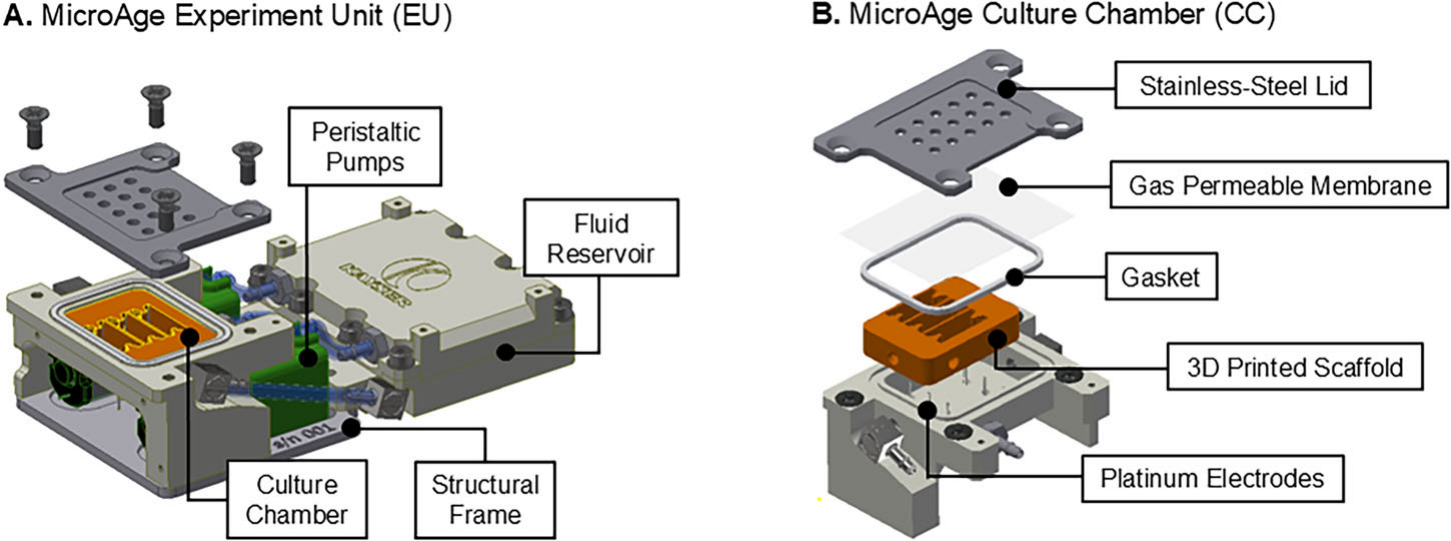
CAD renderings displaying the core components of the Experiment Unit (EU) Design. **A** The EU is composed of the Culture Chamber (CC), fluid reservoir, peristaltic pump assembly and electronics. **B** The CC accommodates the muscle constructs anchored to 3D printed scaffolds. The CC can be further subdivided into its constituent parts, including a stainless-steel lid, gas-permeable membrane, silicone gasket, platinum electrodes and stainless-steel barbs. The whole EU assembly fits within the Experiment Container (EC). Images were produced using AutoCAD.

The MicroAge EU can be broken down into four core subsystems, the CC, reservoirs, electronics and the peristaltic pump assembly (Fig. 3A), a detailed summary of critical EU components is provided in Table 1. The muscle constructs were housed within the CC, anchored to their 3D printed scaffolds. A gas-permeable membrane and vented CC lid permitted gas exchange with both the free air volume of the EC and Kubik, providing an aerobic environment to support cellular respiration (Fig. 3B). The CC was sealed by securing the gas-permeable membrane between a silicone gasket and a stainless-steel lid. The lid featured vent holes and was fixed in place using counter-sunk stainless-steel screws, creating one LoC.

**Table 1:** Details of critical EU components

EU Component	Component details	Manufacturer/Vendor
Peristaltic Pumps	Micro-fluidic peristaltic pump, flow rate 0.45 mL/min. DC geared motor 3 V.	AquaTech Co., Ltd.
Silicone Tubing	Biocompatible silicone tubing, internal diameter 1.5 mm, outer diameter 2.5 mm.	AquaTech Co., Ltd.
Gas Permeable Membrane	Lumox® film, chemically resistant, gas-permeable film.	Sarstedt AG & Co.
Straight and Angled Barbed Fittings	Adjustable 90° elbow fitting, M3 thread to barb. Straight fitting, M3 thread to barb. Stainless steel (316).	Beswick Engineering

The CC body was made from machined PEEK (polyether ether ketone), with fluid handling channels, epoxy cemented platinum electrodes (99.99% purity, 0.5 mm diameter) and mounting points for the pump structural frame. All components of the CC that were in contact with the samples or culture fluids were biocompatible and sterilised by autoclave. During the flight experiment, each muscle construct was electrically stimulated by two platinum electrode pins (six pins per CC), which were attached to a printed circuit board (PCB) underneath the CC. The electrodes were arranged in the CC such that they did not interfere with the sample but were close enough proximity (~1 mm) to electrically stimulate each construct to contract. The CC had a free liquid volume of ~1 mL when the scaffold was in situ.

The fluid reservoir was a two-well design, allowing for the storage of two different liquids when sealed (culture medium and fixative). The reservoir compartments were sufficiently sized so that two medium refreshes and one fixative (PBS or RNALater™) flush could be performed per EU. The culture medium compartment held a total volume of 4.4 mL and the fixative compartment 2.4 mL. Inside the reservoir was a deformable membrane, which served two purposes. Firstly, it allowed for fluids to be pumped in microgravity, acting as a bladder, it forced the fluid towards the outlet. Secondly, it could deform, allowing the waste medium from the CC to be stored in the reverse side of the same well, separated from the fresh fluid. The membrane was designed with a gasket, therefore guaranteeing one LoC.

The EU pump assembly was composed of the peristaltic pumps, tubes, connectors and structural frame, connecting the CC to the reservoir via silicone tubing and stainless-steel barb fittings. Peristaltic pumps were chosen as the optimal system for moving fluids, as the culture fluids only come into contact with the inside of the sterile silicone tubing; they could deliver a flow rate of 0.45 mL/min, and the pumps acted as non-return valves when unpowered, reducing the risk of cross-contamination between the fresh and spent media. The pumps slotted into an aluminium structural frame and were secured to the bottom of the CC using counter sunk stainless-steel screws.

### Electrical stimulation and impedance sensing system

The primary functions of the EU electronics board were four-fold: (a) experiment timeline execution, (b) electrical stimulation of the muscle constructs to contract following a specific contraction protocol, (c) detection of muscle contractions by means of electrochemical impedance spectroscopy and (d) data storage.

The programmed electrical stimulation sequence occurred over a 15-min period and consisted of 180 segments (clusters), lasting 5 seconds each (thus total time of 15 min), delivered by the six platinum electrodes embedded in the base of each CC. Each segment delivered 500 ms of electrical stimulation and a 4.5 s period without stimulation. Within the stimulation period (500 ms), the muscle constructs were exposed to 25 bipolar square wave pulses (1 ms +10 V → 1 ms −10 V) at 50 Hz. These parameters were selected based on extensive prior research using 2D myotube cultures and isolated muscle fibres^22^. The protocol results in one single muscle contraction that lasts ~700 ms (i.e. 500 ms of contraction during the electrical stimulation and ~200 ms allowed for relaxation of the construct).

In the time between stimulation pulses, a low intensity ~200 mV AC signal was used to measure the impedance between the electrodes. One frequency was measured between each stimulation pulse, giving a total 25 impedance measurements (frequency range 1 KHz → 100 KHz). After each stimulation pulse, the system was given 6 ms to stabilise, then 20 consecutive measurements were taken over a period of 2 ms and averaged. The readings were stored in the non-volatile memory. Impedance measurements continued in the 4.5 s between contractions, cycling through one frequency every 20 ms.

The impedance sensing system was validated by performing the stimulation and sensing protocol under controlled conditions. The culture chamber was filled with L15 culture medium and maintained at 37 °C, 5% (v/v) CO_2_. Three experimental configurations were tested: an empty scaffold, a scaffold supporting viable muscle constructs capable of contraction, and a scaffold containing muscle constructs that had been snap frozen, thawed, and rendered non-viable.

### Experiment container design

**Fig. 4:**
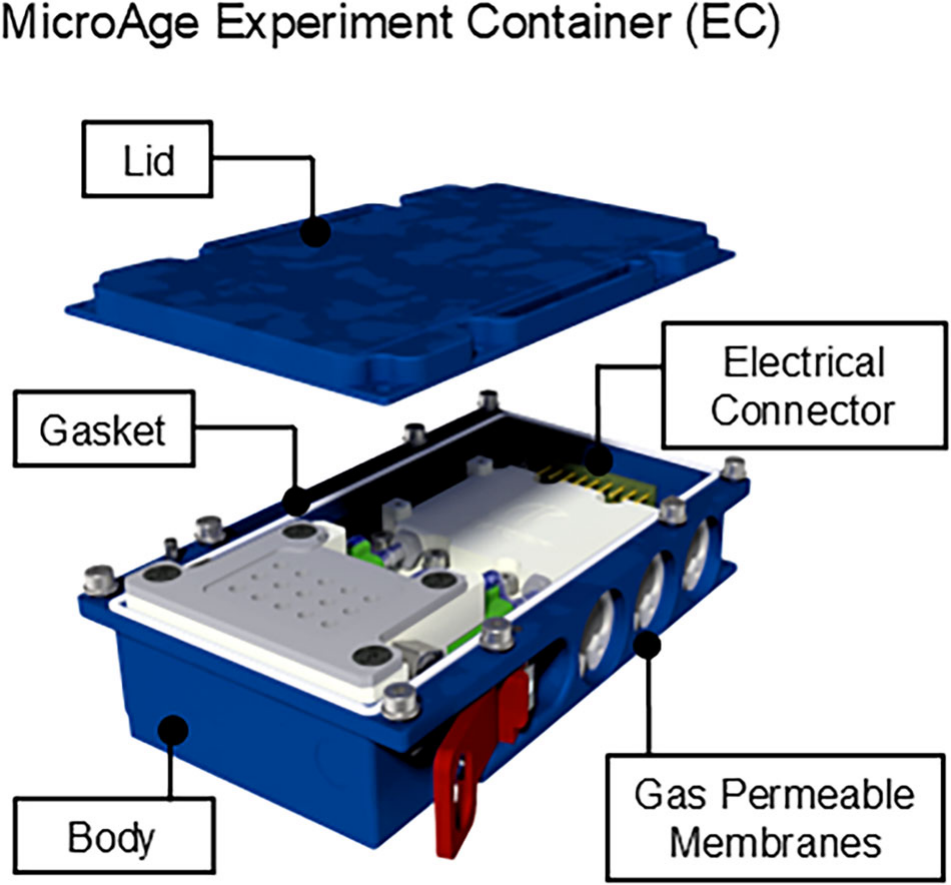
Overview of the microage experiment container (EC), highlighting the constituent components of the design solution. Images were produced using AutoCAD.

The EC was comprised of a lid and body which provided a single LoC for the experiment, with the LoC being maintained through the compression of the silicone gasket by eight screws (Fig. 4). Anodized, high-stress-corrosion and crack-resistant aluminium was used to manufacture the EC to protect it against surface corrosion and cosmetic damage. The interface between the EC and Kubik incubator was via its electrical connector, suppling the EU with power to run the experimental sequence autonomously via firmware.

The design of the EC allowed for free gas exchange with Kubik through three gas-permeable silicone membranes. This method maintains the LoC as the membrane only permits gas molecules to pass. The membrane was sealed to the EC using an ‘O-ring’ and compressed using an elastic, steel ring retainer. To prevent any external damage to the membrane, the assembly is recessed into the EC side wall with a steel mesh plate covering the membrane (Fig. 4).

Temperature data loggers (iButtons – DS1922L) were affixed to the external surface of four ECs in order to monitor the mission temperature profile from handover to sample return.

### Flight hardware integration into Kubik

The MicroAge experiment was specifically engineered around the capabilities and constraints of Kubik, a temperature-controlled, atmospheric CO₂ incubator located on the Columbus module of the ISS. All flight hardware was purpose-built to interface seamlessly with Kubik’s modular design. To meet the experimental objectives, the centrifuge insert (CI) was chosen from Kubik’s range of removable inserts, as it allowed for the simultaneous exposure of 16 experimental containers (ECs) to microgravity and 8 ECs to simulate Earth gravity. The centrifuge operated at 1 g, with the gravity vector oriented perpendicular to the long axis of the muscle constructs. Kubik was pre-warmed to 37 ± 0.5 °C before the experiment commenced and maintained within this temperature range throughout the flight experiment.

### MicroAge experimental design and hardware configuration for flight

This section outlines the structure of the flight experiment, with particular emphasis on how its design was tailored to suit the configuration and operational capabilities of Kubik. Kubik’s modular design and gravity simulation features were central to the organisation and execution of the flight experiment. As displayed in Table 2, the flight hardware was assigned to specific sub-experiments based on several criteria. These included the cell type, either AB1167_CTL or the proof of principle variant that overexpresses HSP10 (AB1167_HSP10), the end point fixative used within each experimental unit, the gravity level to which samples were exposed, which was determined by their spatial arrangement within Kubik, and the electrical stimulation status of the muscle constructs. Each sub-experiment consisted of two experimental units, each containing three muscle constructs, providing six biological replicates for each analytical condition.

**Table 2:** MicroAge experiment unit (EU) groupings

Sub-experiment	Experiment unit no	Cell type	Gravity	Electrical stimulation	Fixation
SE1	**1**	AB1167_CTL	µg	**✓**	RNALater™
	**2**				
SE2	**3**	AB1167_CTL	µg	**✓**	PBS
	**4**				
SE3	**5**	AB1167_HSP10	µg	**✓**	RNALater™
	**6**				
SE4	**7**	AB1167_HSP10	µg	**✓**	PBS
	**8**				
SE5	**9**	AB1167_CTL	1g_s_	**✓**	RNALater™
	**10**				
SE6	**11**	AB1167_CTL	1g_s_	**✓**	PBS
	**12**				
SE7	**13**	AB1167_CTL	1g_s_	✗	RNALater™
	**14**				
SE8	**15**	AB1167_CTL	1g_s_	✗	PBS
	**16**				
SE9	**17**	AB1167_CTL	µg	✗	RNALater™
	**18**				
SE10	**19**	AB1167_CTL	µg	✗	PBS
	**20**				
SE11	**21**	AB1167_HSP10	µg	✗	RNALater™
	**22**				
SE12	**23**	AB1167_HSP10	µg	✗	PBS
	**24**				

*µg* microgravity, *1g*_S_ simulated gravity via centrifugation, *CTL* Muscle constructs fabricated using human immortalised muscle cells, AB1167_HSP10: Muscle constructs fabricated using human immortalised muscle cells overexpressing HSP10.

Upon return of the samples from the ISS, a ground reference experiment was performed, replicating the upload conditions (as recorded by iButton data loggers affixed to the ECs) and the experiment sequence as it occurred during flight. In this scenario, the number of sub-experiments reduced due to the lack of a simulated 1g_s_ condition on the ground. The excess units were used as additional replicates in the AB1167_CTL ± electrical stimulation groups (SE 1–2 and SE 9–10).

### Definition of sample upload conditions

Upload of live, mammalian culture material to the ISS required specific considerations to overcome constraints imposed by the launch and space-flight environments. During the MicroAge mission campaign, access to active temperature control facilities and power were not feasible for the launch and upload phase, as is common for many payloads. As such, culture conditions had to be optimised to ensure that the muscle constructs could survive for a period of ≤120 h using stowage assets with passive thermal control capabilities (Double Cold Bags; (DCBs)), and without access to power to execute autonomous medium refreshes.

Furthermore, neither the launch stowage assets or Kubik could maintain an environmental CO_2_ concentration of 5% (v/v) to provide optimal growth conditions and maintain the pH of the media within a physiological range, as is commonly used for cell culture. This therefore required careful selection of an alternative ‘CO_2_-independent medium’ which was compatible with the cells and did not compromise viability compared with the standard DMEM-based differentiation medium formulation.

### Treatment of myotubes

To screen for suitable media formulations and temperature conditions, myoblasts were harvested via trypsinisation using 1× trypsin-EDTA and seeded as monolayers into appropriate culture vessels. Cells were incubated in complete growth medium for 24 h to support adherence, followed by a 5-day differentiation period in DMEM with GlutaMax™, supplemented with 2% (v/v) horse serum, 10 µg/mL recombinant human insulin, and 10 µg/mL gentamicin.

The differentiation medium was subsequently replaced with one of the following, in accordance with the specified exposure time and temperature conditions of the experiment: (1) DMEM with GlutaMAX™ supplemented with 25 mM HEPES, 2% (v/v) horse serum, 10 µg/mL recombinant human insulin, and 10 µg/mL gentamicin; (2) Leibovitz L-15 medium with GlutaMAX™, supplemented with 5 mM galactose, 2% (v/v) horse serum, 10 µg/mL recombinant human insulin, and 10 µg/mL gentamicin; or (3) Gibco CO₂-independent medium, supplemented with 2 mM L-glutamine, 2% (v/v) horse serum, 10 µg/mL recombinant human insulin, and 10 µg/mL gentamicin.

### LIVE/DEAD™ cell viability assays

Monolayers were washed with Hank’s Balanced Salt Solution (HBSS) before being incubated with SYTO 10 green-fluorescent nucleic acid stain (live) and red-fluorescent ethidium homodimer-1 stain (dead) for 15 min at room temperature. Following incubation, the cells were washed with HBSS before fixation with 4% (v/v) glutaraldehyde. Cells were visualised using a Zeiss Axio Observer Microscope using excitation and emission wavelengths of 483/503 nm (SYTO 10) and 528/617 nm (ethidium homodimer-1).

Data analysis was performed in the ImageJ package Fiji. Images were processed (gaussian blur, thresholding, binary watershed), and the ‘analyse particles’ plugin was used to measure the total area of dead cells and the number of particles (for higher magnification images) within each replicate image per condition. The threshold was kept constant for each experimental dataset. Data were plotted using the RStudio software (Version 2024.12.1 + 563).

## Results

### Bioengineered skeletal muscle constructs

**Fig. 5:**
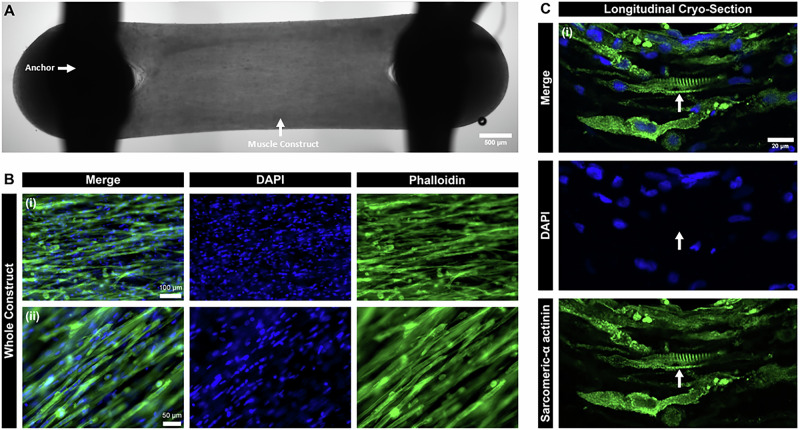
Assessment of skeletal muscle construct architecture. **A** Representative brightfield image of a whole muscle construct fixed in 4% paraformaldehyde (PFA), scale bar 500 µm. **B** Assessment of myotube alignment across the long axis of whole, fixed (4% PFA) muscle constructs. Muscle constructs were stained with Alexa Fluor 488-conjugated phalloidin to highlight the filamentous actin (f-actin) networks in individual myotubes and DAPI to highlight the nuclei, scale bar (i) 100 µm and (ii) 50 µm. **C** Longitudinal cryo-sections of muscle constructs, co-stained to detect sarcomeric-α actinin and nuclei (DAPI), scale bar 20 µm, arrow highlights sarcomeric striations.

Bioengineered skeletal muscle constructs were generated as previously described in Tollitt et al., whereby liquid cell-ECM/fibrin hydrogel suspensions were deposited into the reservoirs of 3D-printed PLA scaffolds^21^. Over 12 days, the constructs compacted around internal anchorage points (Fig. 5A). To evaluate myotube alignment, the entire muscle constructs were fixed in 4% (v/v) paraformaldehyde (PFA) and co-stained with Alexa Fluor (488 nm)-conjugated phalloidin and DAPI to visualise the filamentous actin (f-actin) network within individual myotubes.

Differentiated muscle constructs demonstrated end-to-end alignment of multinucleated myotubes along the construct’s longitudinal axis, as depicted in Fig. 5A, B. Longitudinal cryosections of mature muscle constructs were stained with sarcomeric α-actinin, a structural protein essential for sarcomere stability and organisation^23^, revealing the characteristic striated pattern indicative of the organised arrangement of contractile proteins (Fig. 5C).

### Optimisation of sample upload conditions: CO_2_-independent medium selection

**Fig. 6:**
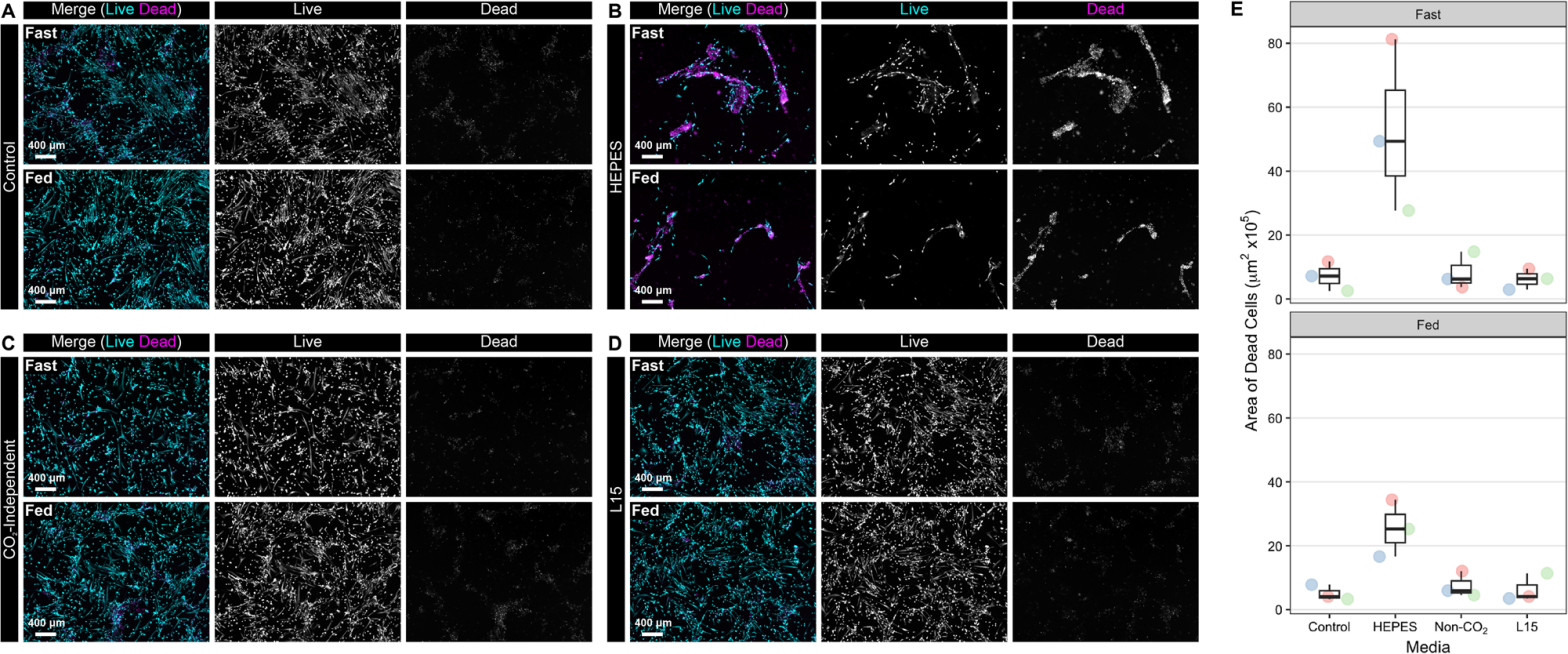
Representative images (*n* = 3) LIVE/DEAD™ assays evaluating the performance of different media formulations, that do not require 5% (v/v) CO₂ to maintain physiological pH, using AB1167_CTL cells over a 120h period (37 °C). Two test conditions were assessed: a ‘fasted’ state without medium exchanges to simulate passive upload conditions and a ‘fed’ state with medium exchanges every 48 h. **A** DMEM-based differentiation medium under standard culture conditions (37 °C, 5% CO₂) was used as the control. **B** HEPES-buffered DMEM with GlutaMax™, supplemented with 25 mM glucose, 10 µg/mL recombinant human insulin, and 2% horse serum. **C** Gibco CO₂-independent medium, supplemented with 2 mM L-glutamine, 10 µg/mL recombinant human insulin, and 2% horse serum. **D** Leibovitz L-15 medium with GlutaMax™, supplemented with 5 mM galactose, 10 µg/mL recombinant human insulin, and 2% horse serum. **E** Illustrates the total area occupied by non-viable cells, identified by ethidium homodimer-1 staining, expressed as µm² × 10⁵ within each field of view. Scale bar: 400 µm. Graphs were generated using R Studio (Version 2024.12.1 + 563).

A series of CO₂-independent medium formulations were screened using AB1167_CTL myotubes in two-dimensional culture to assess performance over an 120h period (Fig. 6B–E). Standard DMEM-based differentiation medium (Fig. 6A) was used as a control (37 °C, 5% (v/v) CO_2_). To simulate a passive upload scenario to the ISS, cells were tested under two conditions: a ‘fasted’ state, with no medium exchanges and a ‘fed’ state, where medium exchanges were performed at regular (48 h) intervals (37 °C, ambient CO_2_). End-point measuresments of cell viability were assessed using a LIVE/DEAD™ cell imaging kit.

The DMEM controls performed as expected, with the myotubes in both the ‘fed’ and ‘fasted’ states remaining viable throughout the culture period. The mean area of ethidium homodimer-1 positive staining (indicating non-viable cells) was 50,899.27 µm² in the fed group and 71,463.11 µm² in the fasted group (Fig. 6A, E). In contrast, myotubes cultured in HEPES-buffered DMEM under ambient CO_2_ conditions exhibited a complete loss of viability, even when medium exchanges were performed at regular intervals (Fig. 6B).

Both the Gibco CO_2_-independent medium (Fig. 6C, E) and Leibovitz L15 medium (Fig. 6D, E) performed well under ambient CO_2_ levels. For the Gibco CO₂-independent medium, the mean area of ethidium homodimer-1–positive staining was 75,439.20 µm² in the fed group and 82,592.61 µm² in the fasted group. In comparison, Leibovitz L15 showed mean areas of 63,528.68 µm² (fed) and 62,490.28 µm² (fasted). Therefore, L15 was selected for use in the mission due to its superior performance.

### Optimisation of sample upload conditions: temperature control

During upload and prior to insertion into Kubik, the ECs were stowed for launch in DCBs. The DCBs are used routinely for passive thermal control of science payloads and are able to maintain a relatively broad temperature range for at least 120 h through the use of pre-conditioned thermal bricks. This was to account for potential launch delays and time to ISS docking.

**Fig. 7:**
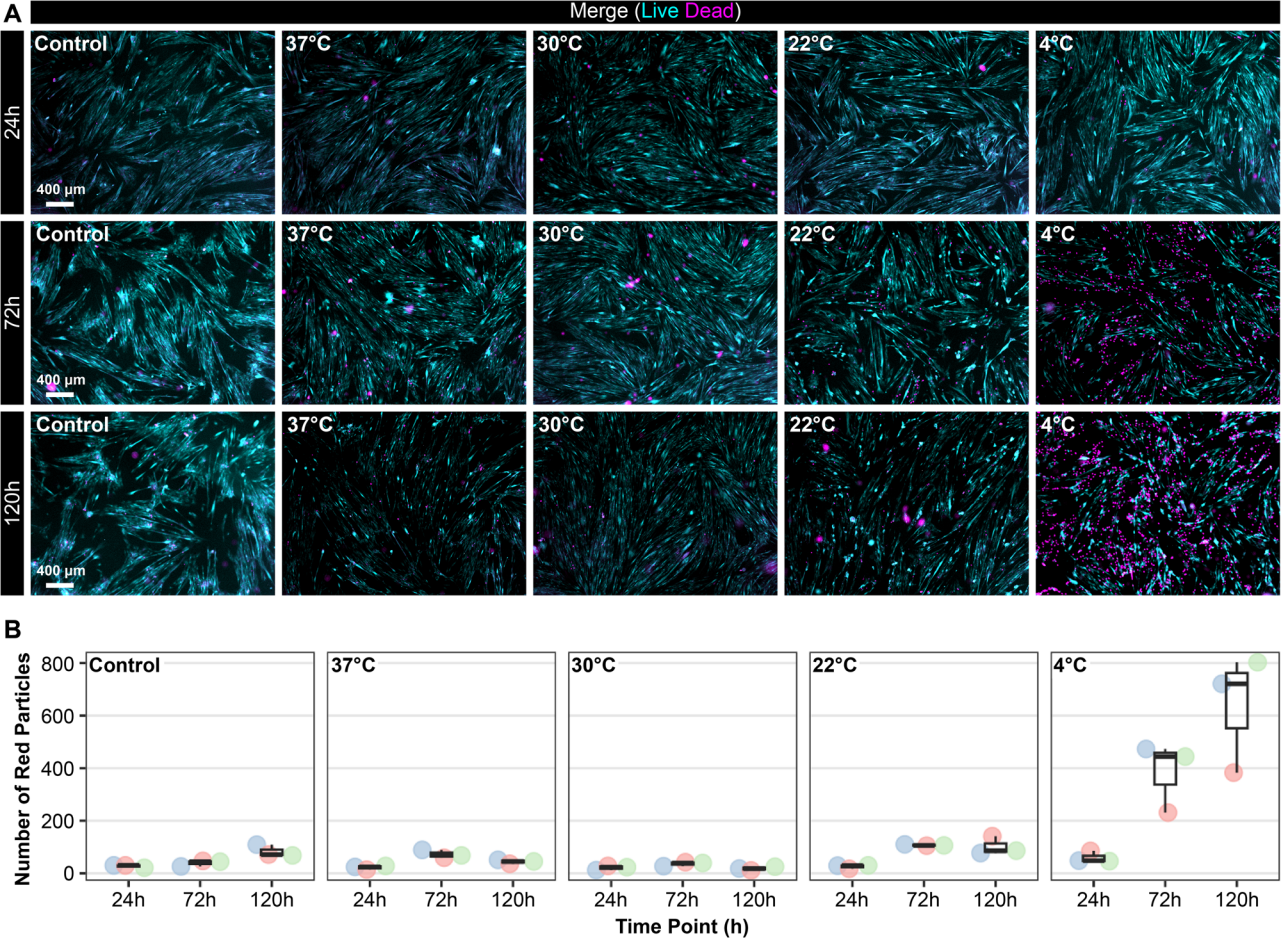
Representative images (*n* = 3) depict temperature tolerance screening of AB1167_CTL myotubes cultured in Leibovitz-L15 medium (ambient CO_2_) at various upload temperature set points (4–37 °C). Cells were incubated over a 120h period without medium exchanges (fasted) to mimic worst-case, passive upload conditions to the ISS. LIVE/DEAD™ assays were conducted at 24, 72 and 120-h intervals to evaluate cell viability. The data were compared to a DMEM control (37 °C, 5% (v/v) CO_2_) across the same culture period, where the medium was refreshed every 48 h (**A**). **B** shows the count of non-viable cells, identified by ethidium homodimer-1 staining and represented as ‘red particles’ within each field of view. Scale bar: 400 µm. Graphs were generated using R Studio (Version 2024.12.1 + 563).

To determine an optimal thermal range for the cells during the upload process, various temperature set points ranging from +4 °C to +37 °C were assessed using Leibovitz L15 medium over the course of 120 h. During this period, medium exchanges were not performed, and AB1167_CTL myotubes were maintained in two-dimensional culture. For comparison, control myotubes were maintained using a standard DMEM-based differentiation medium at 37 °C (5% (v/v) CO_2_), with medium exchanges performed every 48 h. Cell viability was assessed at incremental time points (24, 72 and 120 h) using a LIVE/DEAD™ assay kit. The results are presented in Fig. 7A, B.

The DMEM-based control myotubes, which underwent regular medium exchanges, showed an increase in the number of ethidium homodimer-1 positive cells (i.e. number of red particles) at 120 h. In contrast, myotubes cultured in Leibovitz L15 medium at +4 °C or +22 °C began showing signs of reduced viability as early as 72 h (Fig. 7A, B). More favourable results were observed at +30 °C with myotubes remaining viable across all points tested. Notably, myotubes cultured for 120 h at +30 °C showed a greater number of SYTO10-positive (viable) cells compared to those maintained at +37 °C for the same period (Fig. 7A).

### Validation of the electrical stimulation and impedance sensing system

**Fig. 8:**
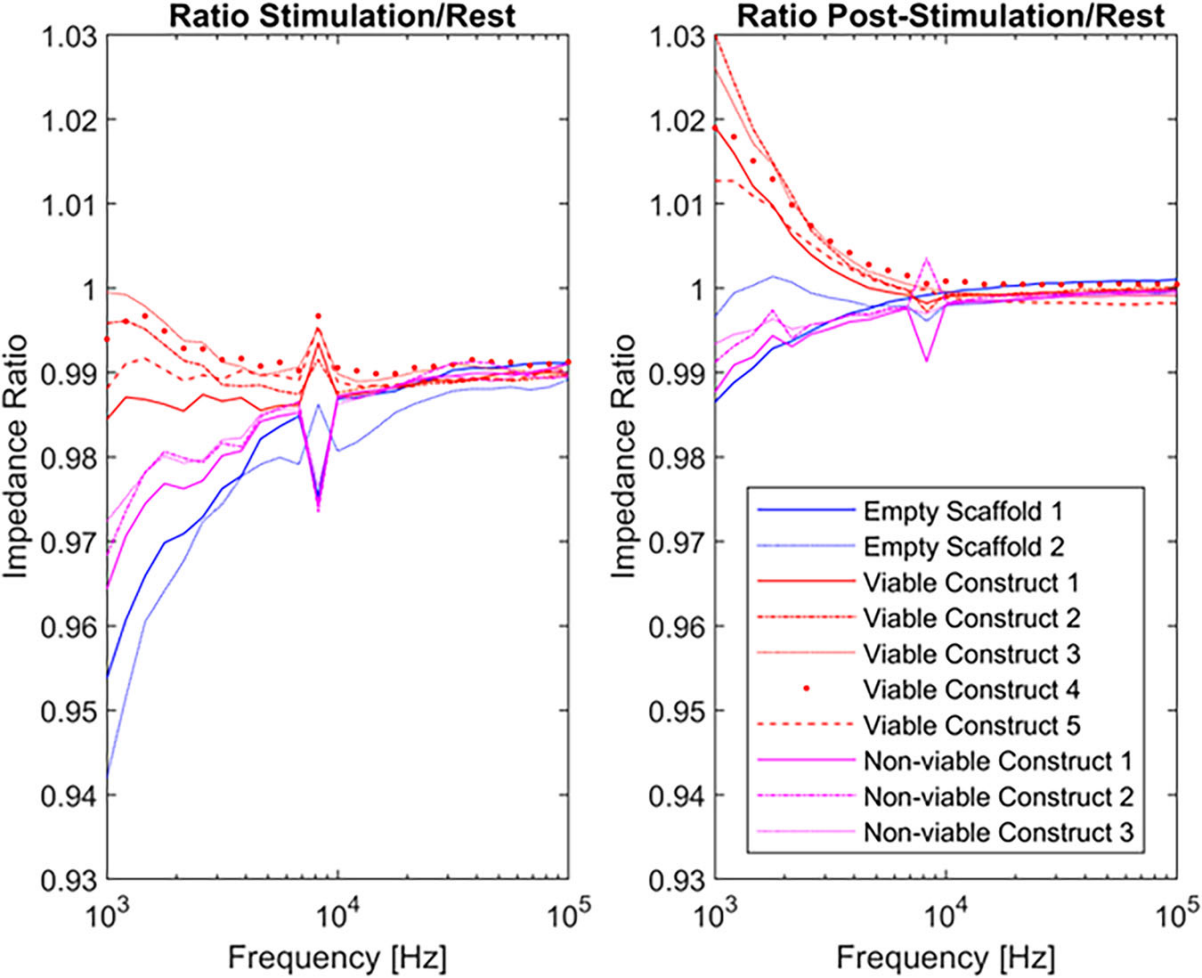
Validation of Impedance Measurements. Measurement of the impedance spectrum of the culture chamber containing either empty scaffolds, viable or non-viable (snap-frozen) AB1167_CTL muscle constructs during the stimulation phase (left) and the post-stimulation phase (right). Graphs were generated using MATLAB 2024a.

The impedance monitoring system continuously measured the impedance spectrum of the CC during the stimulation phase (Fig. 8). Contractions were monitored by calculating the ratio between the impedance measurements during the 0.5 s stimulation phase and the impedance measurements at ‘rest’ (4 s after stimulation). Muscle constructs that do not contract show a flat impedance ratio between the stimulated and rest phases. However, electrical noise from the stimulation signal, due to the formation of an electrical double layer on the electrodes, thus introducing additional capacitance, was distorting the low-frequency response of the system, resulting in a ratio <1 in the region below 10 kHz even without tissue constructs present. However, this divergence was significantly smaller than that observed in viable constructs, underscoring the sensitivity of the method to detect contractile behaviour through spectral analysis.

As a secondary measure, the ratio between the 0.5 s just after stimulation (‘post stimulation’) and the ‘rest’ phase was assessed. Since the impedance changes due to contraction lasts for at least part of the rest period, the signal is still affected by the tissue relaxation, but there is much less electrical interference as the stimulation signal is not present. The ratio between this ‘post-simulation’ region and the rested construct stays close to 1 for all frequencies with empty scaffolds or scaffolds holding non-viable (snap-frozen) constructs. In all cases, the response of contractile (viable) constructs deviated from empty scaffolds or non-viable constructs in the region below 10 kHz that allows for reliable detection of whether contractile activity has occurred. A video of the muscle constructs contracting during the electrical stimulation protocol is provided in Supplementary Fig. 1.

At higher frequencies, the spectral ratio across viable constructs consistently returns to a value of 1, which is consistent with known electrical properties of biological tissues. This behaviour is largely due to the role of cell membranes, which act as capacitive barriers between the extracellular space and the cytoplasm. At lower frequencies, these membranes limit current flow into the cell, making changes in cell shape or structure easier to detect. As frequency increases, the influence of the membrane decreases, and the tissue begins to behave like a uniform material. In this range, small internal changes are no longer detectable. However, including high-frequency measurements provides a useful reference point, helping to confirm system stability and identify artefacts such as trapped air bubbles.

## Discussion

This publication details the extensive design process and preliminary experiments necessary to create hardware and protocols for maintaining the viability and functionality of human skeletal muscle cells aboard the ISS. This work enabled the study of 3D skeletal muscle constructs, derived from human cells, to assess their responses to microgravity exposure and evaluate the influence of overexpressing the mitochondrial chaperone, HSP10. Furthermore, the developed system facilitated investigations into whether muscle constructs on the ISS responded differently to electrically stimulated contractions compared to ground-based reference experiments, and whether inducing artificial gravity (1 g_s_) on the ISS via centrifugation altered microgravity-related responses.

The MicroAge mission data are a timely and valuable addition to the expanding field of research focused on muscle physiology in space. Extensive evidence from spaceflight and bed rest studies has established that microgravity induces rapid skeletal muscle atrophy, largely driven by disruptions in protein balance and metabolic regulation^24^. Yet, it remains uncertain whether microgravity serves as a suitable model of accelerated biological ageing, and whether it can reliably be used to uncover underlying molecular mechanisms and evaluate potential countermeasures for terrestrial benefit^25,26^.

Recent advances using bioengineered muscle constructs, such as those by Kim et al. and Parafati et al., have enabled more precise modelling of these changes, revealing shifts in lipid metabolism, increased apoptosis, and impaired myogenic differentiation in microgravity^27,28^. The MicroAge mission builds on this foundation by integrating engineered muscle tissue into a semi-automated bioreactor system, allowing researchers to examine how electrical stimulation, genetic-interventions and simulated 1g may counteract microgravity-induced muscular decline in one mission campaign.

These findings will help bridge the gap between molecular insights and functional outcomes, advancing the development of targeted countermeasures to mitigate muscle loss during space missions. Importantly, they may also offer translational benefits for ageing populations on Earth, informing strategies to preserve musculoskeletal health across the lifespan.

The pronounced decline in skeletal muscle mass and function seen in humans and animals under microgravity conditions has established skeletal muscle as a primary area of study in this field^29^. These effects are often studied using orbital platforms like the ISS or ground-based microgravity simulation models such as clinostats and random positioning machines^30,31^. Large-scale experiments conducted aboard the ISS have encompassed studies of both human and animal muscle, both prior to and following spaceflight, upon return to Earth^32–34^. Further, there have been numerous studies using cultured muscle cells in various different model systems^35,36^.

Traditionally, these systems have used two-dimensional cultures of mammalian skeletal muscle, derived from satellite cells and driven to differentiate from myoblasts into myotubes adhered to flat, tissue-culture treated surfaces^37^. A great deal of information has been obtained from such models, but the resultant monolayers of myotubes are relatively immature and lack the complex alignment and spatial organisation that reflects native muscle tissue; a limitation that has been increasingly recognised as the field has advanced.

Over the past decade, the biofabrication of 3D skeletal muscle tissue in vitro has garnered significant attention. This advancement has enabled both the cultivation and differentiation of human myoblasts into myotubes, encapsulated within synthetic scaffolds or hydrogels engineered to replicate some of the complexities of muscle extracellular matrix (ECM)^21,38,39^. A comprehensive review of recent trends in skeletal muscle tissue-engineering has been published by Schätzlein and Blaeser (2022).^40^.

For the MicroAge Mission, a cell-hydrogel encapsulation method was selected to develop a tissue-engineered human skeletal muscle model that could be anchored onto bespoke 3D-printed, PLA scaffolds^21^. The scaffolds were designed to hold the three muscle constructs in a fixed position, ensuring that the distance between each construct and the platinum electrodes remained consistent across all CCs. This design was crucial for the reliable and reproducible delivery of the electrical stimulation pulses and detection of the resultant impedance signals. Further, the scaffold design featured microfluidic channels that integrated with the flight hardware fluid handling system, facilitating efficient fluid exchanges (≥90%, data not shown) in the CCs (Figs. 1–2).

An immortalised human skeletal muscle cell line (AB1167_CTL) was chosen to generate the muscle constructs for this study. This cell line was selected due to its extended proliferative lifespan relative to primary human myoblasts, ability to retain fusion characteristics from the parental cell population and its capacity to express a myogenic programme^19,20^. Additionally, the differentiated myotubes derived from this line have been shown to maintain a functional phenotype, capable of spontaneous contractions as well as contractions following electrical stimulation. This makes the cell line an ideal choice for investigating adaptive responses to contractile activity, particularly in the context of space flight^21,41^. A comprehensive summary of the advantages and limitations of the AB1167_CTL cell line is outlined in Tollitt et al.^21^.

As illustrated in Fig. 5, the muscle constructs generated from the AB1167_CTL cell line exhibited highly organised, end-to-end alignment of the myotubes along the longitudinal axis of the construct, enhancing the architectural complexity of the model in comparison to myotubes grown in 2D culture. For the MicroAge mission, a naturally derived ECM-based hydrogel formulation was chosen to enhance cell adhesion and growth. This selection was driven by the presence of naturally occurring bioactive motifs, such as arginine-glycine-aspartic acid (RGD), which play a crucial role in promoting cellular interactions and supporting tissue development^42^. Furthermore, the incorporation of fibrin into the hydrogel formulation introduced binding sites for growth factors, such as basic fibroblast growth factor-2 (bFGF-2)^43^.

A notable limitation associated with the use of ECM-based hydrogels and fibrin arose from their murine and bovine origins, respectively, which complicated proteomic analyses due to sequence homology with human peptide sequences. Moreover, naturally derived ECM-based hydrogels are subject to inherent batch-to-batch variability, presenting challenges in reproducibility. This issue could potentially be circumvented by using synthetic or defined peptide hydrogels^44^. Nonetheless, significant variability persists within biofabrication methodologies for engineering skeletal muscle constructs. Critical parameters under continued investigation and optimisation include hydrogel composition, cellular alignment, cell density optimisation, and maturation protocols. For a comprehensive review, refer to Volpi *et al*.^45^.

In standard cell culture practices, a humidified CO₂ incubator set to 5% (v/v) CO₂ is typically used. The CO₂ dissolves into the cell culture medium, where a fraction reacts with H₂O to form carbonic acid. The carbonic acid subsequently interacts with bicarbonate ions present in the medium, stabilising the pH within a physiological range (7.2–7.4)^46^. As previously discussed, neither the DCB stowage assets for upload nor Kubik could maintain an environmental CO_2_ concentration of 5% (v/v) to provide optimal growth conditions and maintain the pH of the media within a physiological range. Therefore, an alternative CO_2_-independent media buffering system had to be identified and subsequently tested with the cells.

Tests were conducted using three candidate CO₂-independent culture media, with and without regular medium exchanges across a 120h period (Fig. 6). Among these, two media, Gibco CO₂-independent medium and Leibovitz L15 medium, demonstrated favourable performance in the presence or absence of medium exchanges. However, in both cases, L15 slightly outperformed the CO₂-independent medium; therefore, Leibovitz L15 was chosen for use during the mission.

Leibovitz L15 medium was originally formulated for use in CO₂-free systems, buffered by a complement of salts, D-galactose, and free-base amino acids, particularly L-arginine, to maintain a physiological pH in culture^47^. Furthermore, as observed by Chang and Geyer, media supplementation with D-galactose in the presence of pyruvate, α-alanine, and L-glutamine could consistently be substituted with D-glucose without detrimental impacts to cell growth^48^. This finding was later corroborated by Eagle et al., who noted that, in contrast to D-glucose-based formulations, only a small fraction of the D-galactose metabolised by cells was converted to lactic acid, thereby preventing acidification of the medium^49^.

This consideration is particularly critical, as human muscle cells exhibit a propensity for heightened glycolytic activity when cultured in supraphysiological glucose concentrations, compared to muscle tissue in vivo^50^, a phenomenon known as the Crabtree effect^51^. The use of D-galactose and L-glutamine as primary carbon sources forces cells to have an increased reliance on oxidative phosphorylation for ATP production due to negligible net ATP gain from the metabolism of D-galactose^52,53^. The resultant metabolic shift serves additional purposes. Firstly, it renders the cells more susceptible to mitochondrial insult by attenuating compensatory glycolysis^54^. Secondly, it enhances the comparability of the constructs to the metabolic profile of native type I (slow oxidative) muscle fibres.

In scenarios where specific medium formulations are required to sustain cell proliferation or induce differentiation, and a CO₂-free system is not viable, flight hardware can be ‘flooded’ with a defined gas mixture tailored to the needs of the cell type. However, this approach was unsuitable for the MicroAge experiment, as the flight hardware was vented to facilitate gas exchange with the external environment. Alternatively, incubation facilities that have an in-built gas supply are also available on board the ISS^55^.

During the mission campaign, the launch and upload phases were limited by the absence of active thermal regulation and power availability in the launch vehicle, thereby preventing autonomous medium exchanges in the EUs. The lack of medium refreshes was anticipated to result in nutrient depletion and the accumulation of metabolic waste products, potentially compromising the viability of the muscle constructs inside the limited volume of the CC.

The basal metabolic rate of mammalian cells is regulated by a complex interplay of intrinsic and extrinsic factors, with temperature serving as a critical determinant^56^. The potential to reduce the metabolic rate of AB1167 myotubes by lowering the ambient upload temperature was investigated, and this approach aimed to reduce nutrient consumption and preserve cell viability during an extended stowage period (Fig. 7)^57^.

The findings illustrated in Fig. 7 demonstrate that AB1167 myotubes can withstand sub-physiological culture temperatures (30 °C) in Leibovitz L15 medium for up to 120 h without the necessity for medium exchanges. Further, the viability results were either comparable (24–72 h) or exceeded (120 h) those observed with the DMEM-based control. Consequently, thermal bricks preconditioned to +30 °C were selected for upload, as they were reported by NASA to maintain Double Cold Bag (DCB) temperatures between +30 and +25 °C for up to 200 h.

While cellular responses to sub-physiological culture temperatures remain incompletely understood, it is generally acknowledged that such conditions induce cell-cycle arrest and diminished proliferation (where applicable), alterations in mRNA transcription and translation, a reduction in metabolic activity, decreased accumulation of toxic by-products (e.g., lactate), and an overall downregulation of cellular processes^58^. However, the specific temperature range investigated in this study may not be universally suitable for all mammalian cells and experimental parameters should be defined for specific cell types^59^.

Investigating the effect of electrical stimulation on muscle construct responses was a key objective of the project. However, the hardware’s spatial limitations prevented the inclusion of force transducers or an optical system to verify construct contractions after electrical stimulation. Attenuated responses to contractile activity have been suggested as a potential mechanism for muscle atrophy in microgravity, making it essential to confirm that contractions have occurred^60^. It was proposed that geometric alterations of the myotubes within the muscle constructs, both during and following contraction, would result in a detectable change in the impedance of the complex biological material^61–63^.

The system used platinum electrodes to introduce a low-intensity ~200 mV AC signal between stimulations, enabling impedance measurement. The data collected (Fig. 8) demonstrated that the impedance spectrum could effectively differentiate between empty scaffolds, non-viable (snap-frozen) constructs, and actively contracting constructs. As a result, this method was adopted to confirm that contractile activity occurred in the electrically stimulated muscle constructs during the mission.

However, a drawback of the system was that the impedance data represented an average across the entire scaffold, encompassing all three muscle constructs, rather than providing measurements at the level of the individual construct. As a result, it was not feasible to assess the change in impedance of individual muscle constructs within each separate (CC), and therefore, it was not possible to empirically confirm that each muscle construct contracted equivalently.

Lastly, the power limitations of the Kubik incubator rendered the concurrent execution of the electrical stimulation protocol across all relevant units unfeasible (Table 2). As such, the protocol execution was conducted in a staggered manner.

The mission was designed to maximise the utilisation of the Kubik incubator, enabling the accommodation of 72 skeletal muscle constructs on board the ISS. The muscle constructs were arranged three per scaffold across 24 experiment units, providing a minimum of six constructs per experimental group (Table 2). This design strategy was implemented to address a common limitation in microgravity research; the typically low number of biological replicates feasible during orbital missions and/or limited flight opportunities for repeated experimentation^64^. This point also applies to studies of human muscle from astronauts in microgravity, due to the limited number of crew members available on the ISS from which samples can be obtained, in combination with limited public data availability^65^.

While efforts were made to increase replicate numbers, it should be noted that each experimental group (6 constructs) was distributed across only two EUs, with constructs within an EU sharing a common scaffold, culture medium source, and electrical stimulus where applicable. As such, these constitute pseudo-replicates, rather than true independent biological replicates, and this limitation must be considered when interpreting the mission data.

Further limitations relate to the AB1167_CTL cell line itself, which was originally derived from a 20-year-old male donor^19,20,41^. Consequently, the study utilises cells from a single source, restricting the ability to capture inter-individual variation in responses to microgravity, HSP10 overexpression, or electrical stimulation.

As highlighted in the preceding discussion, the AB1167_CTL cell line presented several notable advantages to the study, including ease of use, an extended proliferative lifespan, retention of fusion capacity, and the ability to activate a myogenic programme^19,20^. However, skeletal muscle primary cells and induced pluripotent stem cells (iPSCs) can offer alternative benefits over immortalised human muscle cell lines, particularly with regard to physiological relevance and disease modelling^66^.

Primary cells, sourced directly from human tissue, maintain native gene expression and cellular behaviour, making them well-suited for investigating muscle function and responses to stimuli in a physiologically relevant context. Primary skeletal muscle cell models have successfully flown to the ISS to study the effects of microgravity on muscle function previously^28,37,67^. Meanwhile, iPSCs offer a source of patient-specific cells that can be differentiated into myogenic progenitors, enabling personalised investigations into muscle function. However, further research is needed to overcome challenges such as heterogeneous differentiation, which often results in cell populations with low fusion indices and limited maturity. A comprehensive review of the current literature addressing these issues has been compiled by Iberite et al.^68^.

Both models preserve genetic integrity more effectively than immortalised lines, which are modified to proliferate indefinitely and may exhibit altered behaviour and reduced fidelity compared with native muscle. Although immortalised lines are advantageous for long-term culture and high-throughput applications, they may lack some of the characteristics required for accurate modelling of muscle physiology and pathology.

These preparative studies were completed, and the MicroAge experiment was subsequently launched to the ISS on board the SpaceX 24 cargo resupply mission (CRS) on 21 December 2021, where it was installed into the Kubik incubator onboard the Columbus module. The fixed and frozen samples were returned to Earth ~1 month later.

All data suggested that the hardware functioned nominally, with novel impedance measurements and recovered data, along with biological samples, confirming the model’s suitability for studying the biological effects of microgravity on human skeletal muscle constructs. These findings are currently being prepared for further publication.

## Supplementary information

Supplementary Figure 1 Legend

SupplementaryFigure1

## Data Availability

All data generated or analysed during this study are included in this published article and its supplementary information files.
