# Supplementary material for: MicroAge mission: experimental design and hardware for a bespoke culture system supporting tissue-engineered skeletal muscle
Source: NPJ Microgravity. 2026 Feb 21;12:29. doi: 10.1038/s41526-026-00579-z (PMC13039470; doi:10.1038/s41526-026-00579-z)
Supplement: Supplementary file 1 — Supplementary Figure 1 Legend [file 41526_2026_579_MOESM1_ESM.pdf]

Supplementary Figure 1 Video of a AB1167_CTL muscle constructs contracting during the electrical stimulation protocol on ground.
